# Collecting Hand Wipe Samples to Assess Thirdhand Smoke Exposure

**DOI:** 10.3389/fpubh.2021.770505

**Published:** 2021-12-20

**Authors:** E. Melinda Mahabee-Gittens, Penelope J. E. Quintana, Eunha Hoh, Ashley L. Merianos, Lara Stone, Nicolas Lopez-Galvez, Georg E. Matt

**Affiliations:** ^1^Division of Emergency Medicine, Cincinnati Children's Hospital Medical Center, Department of Pediatrics, University of Cincinnati College of Medicine, Cincinnati, OH, United States; ^2^School of Public Health, San Diego State University, San Diego, CA, United States; ^3^School of Human Services, University of Cincinnati, Cincinnati, OH, United States; ^4^Department of Psychology, San Diego State University, San Diego, CA, United States

**Keywords:** nicotine, secondhand smoke exposure, thirdhand smoke exposure, thirdhand smoke pollution children, hand wipes, dermal exposure

## Introduction

Thirdhand smoke (THS) is the persistent toxic residue of tobacco smoke pollutants that attaches to surfaces, remains in dust, and becomes embedded in environments long after secondhand smoke (SHS) has been released into the air (1). THS pollutants include harmful toxicants such as nicotine, tobacco-specific nitrosamines, polycyclic aromatic hydrocarbons, heavy metals, and other semi-volatile compounds (1). Unlike children's and adult's episodic and variable exposure to SHS, exposure to THS is chronic and may be cumulative over days to years (1–3). Compared to adults, children are more susceptible to inhaling, ingesting, and dermally absorbing THS due to their smaller size, hand-to-mouth behaviors, developing organs, and other varying biological characteristics (4). THS residue from previously smoked tobacco in children's homes or cars creates a pervasive reservoir of THS pollutants on carpets, furniture, clothes, and toys, and in dust (1, 2). Thus, non-smoking children's and adults' overall tobacco smoke exposure (TSE) consists of SHS and THS exposure if they live with tobacco product users or THS exposure only if they do not live with tobacco product users but live in THS-polluted environments (e.g., multiunit homes) (5, 6). Cotinine is a metabolite of nicotine (7), and it can be measured in urine, saliva, blood, hair, and nails (8). Cotinine is the biomarker most frequently measured to assess non-smokers' overall TSE from SHS and THS. However, since cotinine is not specific to THS, cotinine levels cannot be used to determine if non-smokers' TSE includes exposure to SHS and THS or THS alone. Further, cotinine levels are affected by factors other than non-smokers' nicotine exposure such as their age, sex, and genetic factors (7, 9). Although there is no gold standard marker of THS exposure, since THS pollution in non-smokers' environments can be dermally absorbed, nicotine levels on hands serve as a proxy of non-smokers' THS exposure (10–13).

**Figure 1:**
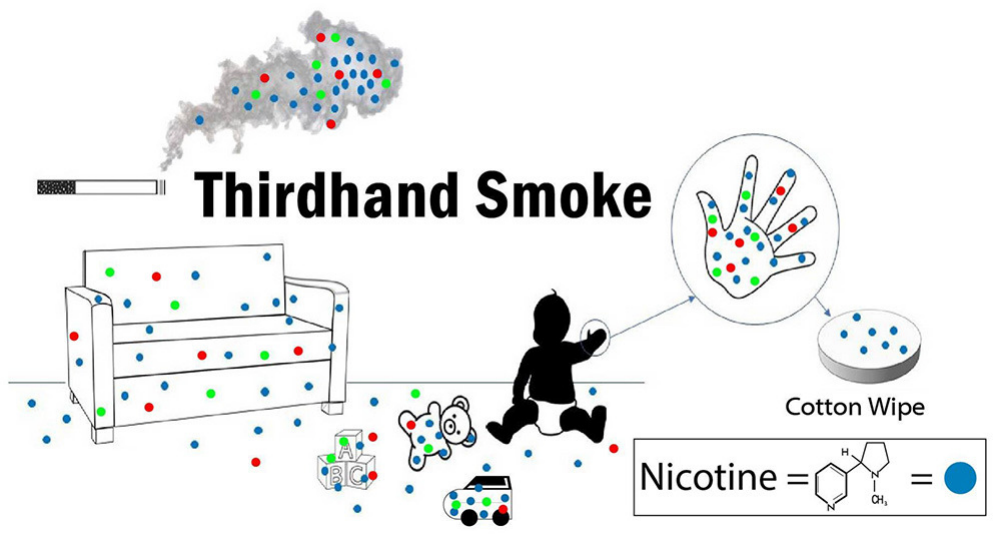
Graphical depiction of thirdhand smoke and hand wipe collection.

We believe that hand nicotine levels are preferable when measuring non-smokers' exposure to tobacco smoke pollutants from THS compared to cotinine (12). Our team was the first to collect and analyze hand wipes from children in the hospital setting (11). Our results indicated that smokers' children bring nicotine, a THS pollutant, on their hands into the hospital setting where no one is smoking. Hand nicotine levels are highly correlated with children's cotinine levels (11–13), and levels may be associated with clinical illnesses (10, 12), but further research is warranted. Clinical researchers who are studying the health effects of child or adult TSE and ways to reduce or eliminate TSE should consider obtaining hand wipes as a way to assess children's or adults' THS exposure. The inclusion of hand nicotine levels in TSE research will help determine: (1) the prevalence of THS exposure in non-smokers; (2) the associations between THS exposure and clinical illnesses; (3) sources of THS pollution that should be targeted in remediation efforts; and (4) sociodemographic differences in THS exposure patterns and potentially modifiable risk factors (e.g., lack of smoking bans) that can be targeted in TSE interventions (see Figure 1).

In this paper, we present methods that represent best practices to collect hand wipes from child or adult research participants in clinical and non-clinical settings. Recommended quality control/quality assurance practices that should be used are also presented to ensure the samples are collected correctly and that the measurements obtained represent reliable and valid nicotine levels.

## Materials and Methods

### Preparation for Hand Wipe Collection

#### Pre-screening Wipes

Wipes must be prescreened by the clinical research coordinator (CRC) before use. Purchase 100% cotton facial wipes (dimensions 9.5 × 6.7 × 3.9 inches; 2.56 ounces; e.g., 80 pack of Swisspers Cotton Organic Rounds). For each purchase, select 10% from the pack of wipes and measure for nicotine with isotope-dilution liquid chromatography with tandem mass spectrometry (LC-MS/MS) (14) with a limit of detection (LOD) at 0.19 nanogram (ng)/wipe (12). Acceptable levels of contamination in each wipe screened from the batch are <1 ng/wipe. If contamination levels in the screened sample wipes are higher than that, discard the batch and repurchase wipes.

#### Baking Amber Sample Collection Vials

Wash vials with water and detergent. Then, cover the mouths of the vials with aluminum foil, place in a laboratory oven, and bake at 450°C for 6 h. Rinse Teflon caps with methanol three times, let dry, and close the vials with cleaned Teflon caps affixed to them.

#### Hand Wipe Sampling Kit Assembly

Sampling kits are prepared prior to use in a clean area while wearing gloves. For each hand wipe sample planned, the kits should contain:

1. Gloves (acrylonitrile), ≥ 2 pairs
2. One piece of 12" × 18" aluminum foil
3. Two 100% pre-screened cotton facial wipes (see section Pre-screening Wipes above)
4. Two polystyrene petri dishes [diameter: 100 mm, height: 15 mm; FisherScientific Catolog (FisherSci Cat) #: FB0875712]
5. Two 2 milliliter (ml) sterile plastic disposable transfer pipettes (FisherSci Cat #: 137119D)
6. Two 20 ml Environmental Protection Agency Screw amber glass vials with Teflon-lined caps (FisherSci Cat #: 03-377-36)
7. One 15 ml sterile polypropylene centrifuge tube with 10 ml distilled water (FisherSci cat#: 07-201-330)
8. One 15 ml sterile polypropylene centrifuge tube with 0.10 grams ascorbic acid (FisherSci Cat # 50-213-151), weighed out on a microbalance
9. Tube rack
10. One-gallon self-sealing plastic bag.

### Hand Wipe Sample Collection

#### Preparation of Wipes

Follow these procedures up to 48 h prior to hand wipe sample collection. At the end of this preparation, CRCs will have a petri dish with two wipes ready to deploy and sample collection vials readied for use if these steps are followed:

1. In a clean work area out of reach of children, set up the sample material workstation by placing aluminum foil on this clean surface.
2. Put on a new pair of gloves and put the tube of distilled water and the tube with ascorbic acid side-by-side in the rack and loosen the caps. Remove a transfer pipette from the wrapper and hold it in the dominant hand and with the non-dominant hand, remove and hold the cap from the water tube. Draw 1 ml of distilled water into the pipette. Open the ascorbic acid tube, hold the cap in the non-dominant hand, and squeeze distilled water into the tube. Repeat these steps until there are 10 ml of distilled water in the tube with the ascorbic acid. Replace and tighten the cap and invert the tube repeatedly to completely dissolve the powder.
3. Unwrap another transfer pipette, open a petri dish with the two cotton rounds, and place the lid on the table, open side up. Using the pipette, squirt 1 ml of ascorbic acid solution onto one cotton round; repeat with an additional 1 ml of solution. Repeat steps to squirt each cotton round with a total of 2 ml ascorbic acid solution; wipes are stable for at least 8 h.
4. Remove two amber vials from the plastic bag, place on the table, remove the aluminum foil from the amber vial, discard the foil, and retighten the lids. Using a permanent marker and pencil, label one of the vials as “blank” and the other as “hand wipe” with a number and date that matches the sample number of the hand wipe sample.

### Obtaining the Hand Wipe Samples

Before entering the participant's room, the CRC will need the prepared wipes and sample vials, gloves, a data sheet to record sample collection, signed informed consent documents as needed, and a questionnaire.

#### Blank Collection

Put on a new pair of gloves, set the amber vial labeled as “blank” on the table, remove the Teflon cap, and set the cap face up on the table. Open the petri dish, place the cotton round into the amber vial, replace the cap, and place the vial in the plastic bag.

#### Hand Wipe Sample Collection

Set the amber vial labeled “hand wipe” on the table, remove the Teflon cap, and set the cap face up on the table. Grasp the wipe firmly in the gloved hand and prepare to obtain the hand wipe sample from the participant's dominant hand, or, if the participant is too young to determine the dominant hand, the right hand is used. Sample the palm of the hand and volar aspect (inward facing part) of all fingers. Wipe from the wrist to the base of the fingers (knuckles). Use five strokes, starting from the base. Next, wipe the fingers and thumbs, including the finger web areas and sides of the fingers, followed by the fingertips. Place the cotton round in the amber vial, replace the cap, and place the vial in the plastic bag.

#### Hand Measurement

Obtain the participant's hand measurements using measurement digital calipers (e.g., Adoric 0-6" Calipers Measuring Tool—Electronic Micrometer Caliper). Measure from the base of the palm to the tip of the middle finger (length) and from the width of the palm without including the thumb; record measurements in centimeters on the data collection sheet.

#### Questionnaire

In the questionnaire, ask the caregiver, child, and/or adult participant the last time that hand washing occurred (including what was used, e.g., soap and sanitizer) and/or the last time that lotion or any other products were put on the participant's hands; record the information on the data collection sheet.

#### Labeling, Storage, and Shipment

Record the date and time of hand wipe collection and sample numbers and submerge the plastic bag containing the samples in a cup of ice. Collected samples and field blanks should be placed on ice in a cooler and transported as quickly as possible (within 4 h) to a freezer where they can be stored at ≤ −20° Celsius until shipment and/or analysis. Ship samples and blanks on dry ice *via* overnight mail to the laboratory for analysis. Nicotine analysis of hand wipes by isotope dilution LC-MS/MS has previously been described (14).

## Special Considerations For Busy Pediatric Clinical Settings

Collecting hand wipes during clinical visits can be challenging but feasible with planning. To facilitate hand wipe collection, the CRC should have all materials and collection vials prepared and labeled before entering the participant's room. It is important to clean the surface that will be used to set up the collection materials and place the wipes as far from caregivers, children, and/or adult participants as possible. The CRC should try and make the collection procedure a fun “game” for children by asking them to help count their fingers and other tactics. Although caregivers may wish to help with the sample collection, it is important to avoid this as parents may have nicotine on their own hands. Ideally, hand wipes should be collected prior to the child washing or sanitizing their hands. This may be difficult when frequent hand washing and the use of hand sanitizers are being employed to decrease the spread of the severe acute respiratory syndrome coronavirus-2 (SARS-CoV-2), the virus which causes coronavirus disease 2019 (COVID-19) (15); thus it is important to ask parents what hand hygiene methods have been used prior to hand wipe collection.

## Reporting Hand Nicotine Levels

Field blank values (ng nicotine) should be subtracted from the hand wipe sample values in ng before reporting results. Analyzing field blanks that are equal to ≥20% of the total sample size is acceptable. The average of the analyzed field blanks in ng nicotine should be calculated for each analysis batch and the average value subtracted from hand wipe sample results. Field blank values may be higher for children with TSE than those unexposed (16). However, if field blank levels exceed 5 ng/wipe, investigators should review data collection practices, transportation, sample handling, and sample analysis to consider changes to reduce sample contamination. Results are reported in ng per hand wipe or ng per m^2^ if hand surface area is adjusted for when hand measurements are available.

## Sample Ranges of Hand Nicotine and Field Blank Levels on Children of Tobacco Product Users in Research

In a previous study (12), hand wipe samples were collected from 276 pediatric emergency department patients [Median (Mdn) age = 4.0 years] who lived with at least one adult who smoked cigarettes. All children had detectable hand nicotine levels ranging from 4.0 to 2,191.3 ng/wipe; Geometric Mean = 89.7 ng/wipe; 95% Confidence Interval = (78.9; 102.0), Mdn = 102.6 ng/wipe, Interquartile range = 46.4–181.9. Field blanks were analyzed to adjust for potential contamination of the wipe samples; the Mdn for the field blanks was 1.8 ng nicotine/wipe and field bank corrections ranged from 0 to 16.7 ng nicotine/wipe for the 276 children.

## Summary

This paper provides a collection method for obtaining hand wipes in children or adults that can later be analyzed for nicotine, a THS pollutant. By obtaining field blanks to adjust for potential environmental contamination with nicotine, accurate measures of hand nicotine levels can be obtained. Assuring complete contents of sample collection kits, detailed directions for sampling participants' hands, and immediate sample storage at ≤ −20°C will facilitate proper collection and ensure sample integrity. The methods described can be translated to pediatric and adult populations in a variety of settings including clinical settings such as the emergency department, urgent care departments, and hospitals, as well as non-clinical settings such as homes and workplaces. We strongly recommend obtaining hand wipe nicotine levels in clinical research studies to obtain a more complete picture of children's and adults' overall TSE.

## Author Contributions

EM-G and GM conceptualized the article, drafted the manuscript, and approved the final manuscript as submitted. PQ, EH, AM, LS, and NL-G drafted the manuscript and approved the final manuscript as submitted. All authors contributed to the article and approved the submitted version.

## Funding

This work was supported in part by the National Institute of Environmental Health Sciences (NIH Grant Numbers R01ES030743, R01ES027815, and R21ES032161), and the National Institute on Drug Abuse (NIH Grant Number K01DA044313).

## Conflict of Interest

The authors declare that the research was conducted in the absence of any commercial or financial relationships that could be construed as a potential conflict of interest.

## Publisher's Note

All claims expressed in this article are solely those of the authors and do not necessarily represent those of their affiliated organizations, or those of the publisher, the editors and the reviewers. Any product that may be evaluated in this article, or claim that may be made by its manufacturer, is not guaranteed or endorsed by the publisher.
